# Dissecting pain processing in adolescents with Non‐Suicidal Self Injury: Could suicide risk lurk among the electrodes?

**DOI:** 10.1002/ejp.1793

**Published:** 2021-05-31

**Authors:** Caterina Leone, Serena Galosi, Cristina Mollica, Mattia Fortunato, Chiara Possidente, Valeria Milone, Sofia Misuraca, Luana Berillo, Andrea Truini, Giorgio Cruccu, Mauro Ferrara, Arianna Terrinoni

**Affiliations:** ^1^ Department of Human Neuroscience Sapienza University Rome Italy; ^2^ Department of Methods and Models for Economics, Territory and Finance Sapienza University Rome Italy

## Abstract

**Background:**

Although non‐suicidal self‐injury (NSSI) disorder is highly prevalent in adolescents, its relationship with pain system function and suicidality is still controversial. The present study was designed to assess the function of the nociceptive afferent pathways and the endogenous pain modulation in adolescent patients with NSSI and to longitudinally register any suicide attempt, describe its frequency and find a possible association between suicide, neurophysiological measures and psychological measures.

**Methods:**

We enrolled 30 adolescents suffering from NSSI and 20 age‐ and gender‐matched healthy controls. Patients underwent a comprehensive psychological evaluation. Each participant underwent thermal pain thresholds of the quantitative sensory testing, laser‐evoked potential recording to study the ascending nociceptive pathway and the conditioned pain modulation testing to test the endogenous pain modulation.

**Results:**

We found that patients with NSSI had a reduced amplitude of the N2 component of laser‐evoked potentials and an abnormal conditioned pain modulation. The amplitude of the N2 was associated with suicidal risk.

**Conclusions:**

The deficit of the endogenous pain modulation likely depends on a saturation due to continuous pain solicitation. The strong association of a reduced amplitude of the N2 component with suicide suggests that it may serve as a possible biomarker in self‐harming adolescents.

**Significance:**

The present study identifies the N2 component of laser‐evoked potentials as a possible neurophysiological biomarker of suicidal risk in patients with non‐suicidal self‐injury, therefore, raising the possibility for a non‐invasive test to identify subjects at higher risk of suicide among self‐harming patients.

## 1 INTRODUCTION

Non‐suicidal self‐injury (NSSI) is broadly defined as a direct, not socially sanctioned behaviour that causes physical injury which results in the destruction of one's own body tissue in the absence of any observable intent to die (Muehlenkamp, 2005). In recent years, the increase in Western countries of self‐injurious behaviours, especially during adolescence and young adulthood, has made NSSI a major public health issue (Klonsky, 2011). Because NSSI can occur alone or in comorbidity with a range of disorders, such as major depressive disorder (MDD; Hintikka et al., 2009), borderline personality disorder (BPD; Cerutti et al., 2011), eating disorders (Claes et al., 2018) and other disorders (Apter et al., 2008; Chart rand et al., 2012; Fliege et al.,^2009^; MacLean et al., 2011; Serras et al., 2010), the ‘DSM‐5’ proposal is now recommending to consider NSSI as an independent condition in need of further study. Since a few studies focused on the neurobiology of the disease in adolescents and its clinical course, the pathogenesis is controversial, particularly its link with pain processing, personality disorders and suicidality (Westlund Schreiner 2015) (Griep & MacKinnon, 2020).

The common reporting of a reduced or absent pain perception in NSSI patients during self‐harming partially answers the legitimate question on how these patients can overcome the instinct to avoid pain and raise the possibility of an abnormal pain system function, as described in previous studies on self‐harming adults (Franklin et al. 2012; Magerl et al. 2012; Schmahl et al. 2004). Given the lack of results on adolescents with recent diagnosis of NSSI not associated with BPD, it is not clear whether the pain system abnormality is present since disease onset, or rather should be attributed to the severity and duration of the disease, or to the association with a major psychiatric disorder. Recent studies pointed to the role of pain and emotion dysregulation in the transition from suicidal thoughts to action (Conejero et al.2018; Kim et al. 2020), suggesting a possible association between pain processing and suicide. Although a large collection of psychological variables have been associated with suicide progression (Grandclerc et al.2016; Victor & Klonsky, 2014), no information is available in regard to quantitative neurophysiological measures.

We, therefore, enrolled a population of adolescents with NSSI and followed up our patients for 2 years, setting up the study in two parts and gaining information on pain and suicidality. We performed a cross‐sectional neurophysiological study of a homogeneous population of adolescents with NSSI versus a control population aiming to: (1) assess the integrity of the pain system in terms of thermal pain thresholds, laser pinprick thresholds, laser‐evoked potentials and endogenous pain modulation and (2) find a possible correlation between the above‐mentioned parameters and the severity and duration of the disease. We then longitudinally a) described the prevalence of suicide in our population, b) identified two distinct populations of patients (suicidal and non‐suicidal) to compare their neurophysiological and psychological data, collected in the first part of the study and c) assessed the presence of any predictive factor for suicidality among the variables recorded during the study.

## 2 MATERIALS AND METHODS

### 2.1 Study cohort and design

We consecutively screened 42 patients and enrolled 30 patients (12 patients and/or their guardians declined to participate) with NSSI diagnosed within 5 years from the symptoms onset (age 11–18 years, mean 15.7; 27F) with five or more self‐injurious behaviours without suicidal intention occurred in the last year. Patients were consecutively recruited among the inpatients of the Adolescent Unit of the Child Psychiatry Department of the ‘Sapienza’ University. As a standard assessment procedure, all subjects were interviewed and screened for DSM‐5 diagnoses with the Italian version of the Schedule for Affective Disorders and Schizophrenia for School‐Age Children/Present and Lifetime Version (K‐SADS‐5: Townsend et al. 2019 Italian version) and diagnoses were distributed as follows: depressive disorders (11 patients), anxiety disorders (8), bipolar disorder (4 patients), behavioural disorders (4 patients), eating disorder (3 patient). Exclusion criteria were peripheral or central nervous system disorders, bipolar disorder type 1, schizophrenia spectrum disorders and neurodevelopmental disorders. At the time of investigation, the majority of the NSSI patients were assuming drugs in stable regimen: quetiapine (*N* = 9), aripiprazole (*N* = 6), selective serotonin reuptake inhibitors (*N* = 4), no drugs (*N* = 11).

All patients underwent a comprehensive psychopathological evaluation for NSSI assessment, mood characterization and personality dimension assessment.

For the neurophysiological evaluation, a control population of 20 healthy subjects matched for age and sex (age 11–18 years, mean 16.8; 18F) was recruited among patients’ friends and relatives.

Each subject underwent a comprehensive evaluation of the pain system function by (1) static psychophysical measures of sensory and pain thresholds for the nociceptive system evaluation, using quantitative sensory testing (QST) and laser‐evoked potentials (LEP) and (2) dynamic psychophysical measures to evaluate the endogenous pain modulation, using the conditioned pain modulation (CPM) testing.

All procedures took place at the Department of Human Neuroscience of Sapienza University Rome, in accordance with the Declaration of Helsinki. This research was approved by the local institutional review board (Prot. n 278/16 RIF.CE: 4007) and all patients and their guardians gave their informed consent.

### 2.2 Psychopathological instruments

Each NSSI patient was evaluated with the following psychopathological measures, extensively described in previous works: Deliberate Self‐Harm Inventory (DSHI) (Gratz, 2001), Repetitive Non‐Suicidal Self‐Injury Questionnaire (R‐NSSI‐Q) (Manca et al. 2014) and Clinician‐rated severity of non‐suicidal self‐injury (CRS‐NSSI) for NSSI assessment (Somma et al. 2019); Child Depression Inventory (CDI) (Kovacs, 1985) and Beck Hopelessness Scale (BHS) (Beck et al. 1989) for mood characterization; Personality Inventory for DSM‐5 (PID‐5) for personality dimensions assessment (Krueger et al. 2012; Somma et al. 2019).

### 2.3 Thermal pain thresholds with quantitative sensory testing

For quantitative sensory testing, we used a thermode (ATS, PATHWAY, Medoc, Israel). The computer‐driven PATHWAY system contains a metal contact plate (contact area 30 × 30 mm) equipped with an external Peltier element that cools and heats the plate to target levels. The baseline temperature of 32°C reached target temperature at a ramp rate of 1°C/s. Quantitative sensory measures were tested on the right‐hand dorsum. In all patients, we tested the cold detection threshold (CDT), the warm detection threshold (WDT), the cold pain threshold (CPT) and the heat pain threshold (HPT). All procedures took place in accordance with the recommendations given by the German Research Network on Neuropathic Pain (Rolke et al. 2006).

### 2.4 Laser‐evoked potentials

To study laser‐evoked potentials, we used a neodymium: yttrium‐aluminium‐perovskite (Nd:YAP) laser (wavelength 1.34 mm, pulse duration 2–20 ms, maximum energy 7 J) (Di Stefano et al. 2017). The dorsum of the right hand was stimulated by laser pulses (intensity, 89–140 mJ/mm2, 2x individual pinprick threshold; duration, 5 ms; diameter, 5 mm) eliciting pinprick sensations. The interstimulus interval was varied pseudorandomly (10–15 s). After each stimulus, the laser beam target was shifted. We determined the laser perceptive thresholds by delivering stimuli in series at increasing and decreasing intensities and defined the perceptive threshold as the lowest intensity at which the subjects perceived at least 50% of laser stimuli and the pinprick threshold as the lowest intensity at which the subjects perceived a clear pinprick sensation. We asked subjects to mentally count the stimulations perceived in order to maintain steady vigilance/attention (Cruccu 2008). We therefore asked subjects to rate the pinprick threshold on a numerical rating scale (0–10). The EEG was recorded using 32 Ag–AgCl scalp electrodes placed according to the International 10–20 system, referenced to the nose, to allow the recording of the negative–positive complex maximal at the vertex, named N2‐P2 (recorded at Cz referenced at the nose), preceded by a smaller temporal component named N1 (recorded at T5 referenced at Fz).

Electroculographic (EOG) signals were simultaneously recorded using surface electrodes.

The EEG data were preprocessed using Letswave, a free signal‐processing toolbox developed in Delphi 7.0. (https://www.letswave.org). Continuous EEG data were bandpass filtered, with a Butterworth filter, from 1 to 30 Hz for analysis in the time domain. EEG epochs were extracted using a window of 1,500 ms (−500 to 1,000 ms relative to stimulus onset) and baseline corrected using the prestimulus interval. Trials contaminated by eye blinks and movements were corrected using an independent component analysis algorithm (Delorme & Makeig, 2004) Epochs with amplitude values exceeding ±100 µV were rejected. After baseline correction (reference interval: −500 to 0 ms), the data were re‐referenced to Fz (in different data sets). Separate average waveforms were computed for each participant.

### 2.5 Conditioned pain modulation

To test the CPM, we used the CPM‐Sense‐Q: Model, 2001‐TSA, Analyzer Sensory T (Medoc, Israel), consisting of two 30 × 30 Peltier thermode, one used as test stimulus and the other as conditioning stimulus.

CPM was performed using a parallel paradigm (Leone et al. 2019). The noxious ‘test stimuli’ were delivered before, and then simultaneously with, the noxious ‘conditioning stimuli’. Contact heat applied to the non‐dominant volar forearm (dermatome C5, C6) served as the ‘test stimulus’. The intensity of the test stimulus was predetermined individually for each participant, based on the psychophysical parameter of ‘pain‐60’ (Granot et al. 2008). Using the method of limits, with a baseline temperature at 32°C and an increase rate of 2C°/s, we asked each subject to press a stop button as soon as they perceived a painful sensation with an intensity of at least 60 on a 0–100 NRS scale for three consecutive trials. The average of the three trials was considered the target temperature (pain‐60) for the test stimulus.

After 15 min, we performed the CPM session. First, using the ramp and hold method, we delivered two consecutive heat stimulations using ‘pain‐60’ as target temperature to the non‐dominant forearm. Then, we delivered to the dominant forearm (dermatome C8, T1) a conditioning heat stimulus lasting 60 s, using the same stimulation temperature of the test stimuli. During the last 30 s of this conditioning stimulation, two “test stimuli” were repeated consecutively on the non‐dominant forearm, and their intensity rated. The CPM effect was calculated as the difference in the average pain scores of the mean rating of the conditioned and unconditioned ‘test stimuli’. A negative value indicates intact CPM (Yarnitsky et al. 2012).

### 2.6 Statistical methods

As descriptive summaries of the sample composition, we adopted percentage frequencies for categorical variables and mean ± *SD* and median with IQR for quantitative variables.

A preliminary analysis of association was performed by evaluating group differences (controls versus. NSSI cases and attempted vs. non‐attempted suicide) with the Mann–Whitney test or unpaired Student's *t* tests for continuous predictors and with chi‐squared or Fisher's exact test for categorical ones and the analysis of variance (ANOVA) between subgroups (controls, suicide attempt and non‐suicide attempt) with post hoc multiple comparisons and false discovery rate (FDR) correction.

Moreover, since we considered a wide set of explanatory variables, capturing multiple aspects of participants’ clinical condition that can possibly affect the outcome of interest, the predictors which contribute to measure common traits of the individual status were naturally expected to form clusters of highly correlated factors. This phenomenon, known as multicollinearity, imposed the adoption of variable selection criteria to avoid information redundancy and unreliable estimates of the predictive value of the single covariates (Vatcheva et al. 2016) (supplemental material).

The results of the exploratory association study were used as a preparatory step for a more accurate multivariate analysis, aimed at inferring the factors characterizing the suicide attempt in a reduced set of selected potential predictors, through the specification and estimation of a logistic regression models with suicide attempt as the binary outcome variable (Tolles & Meurer, 2016). The final logistic regression model with the optimal set of predictors was determined through the application of the backward selection technique, relying on the Akaike information criterion (AIC) (Akaike, 1974). Finally, we built the receiver operating characteristic (ROC) for the significant covariates and for the entire logistic model and quantified the corresponding diagnostic performance in correspondence of the optimal cut‐off.

All tests performed in the analysis were two‐sided and a *p*‐value <0.05 was considered statistically significant. The statistical analysis was performed with the R software (R Core Team 2020).

## 3 RESULTS

### 3.1 Study cohort

The average age was 15.7 years (11–18) for patients and 16.8 years (11–18) for controls. The average duration of illness was 19.9 months (1–60).

Within 2 years, 16 patients attempted suicide (53.3%).

### 3.2 Pain system evaluation: Patients versus controls

Results from univariate analysis are summarized in Table 1.

**TABLE 1:** Univariate analysis of neurophysiological data for patients versus controls

	Patients (30)	Healthy subjects (20)	*p* value
CDT (˚C from baseline)	4.5 (5.06) 2.7 (1.1;6)	3.6 (2.3) 3.1 (1.62;5.1)	0.399
WDT (˚C from baseline)	3.8 (3.04) 2.7 (1.7;4.75)	3.13 (1.77) 2.75 (1.9;3.75)	0.316
CPT (˚C)	9.05 (8.52) 6.9 (2;17)	10.83 (8.79) 9.35 (4;16.28)	0.487
HPT (˚C)	46.51 (3.01) 46.8 (43.25;49.45)	45.3 (4.34) 45.9 (43.85;48.75)	0.287
Laser Pinprick Thr. (mj/mm^3^)	82.23 (18.49) 76 (73;89)	80.7 (15.1) 76 (76;89)	0.750
NRS Pinprick (0–10)	3.42 (1.7) 3 (2;5)	3.25 (1.5) 3 (2;4)	0.715
LEP N1 Amplitude (µV)	6.27 (5.5) 4.4 (2;9)	6.74 (5.14) 6.65 (2.25;7.75)	0.769
LEP N2 Amplitude (µV)	16.75 (10.51) 14.5 (7.75;23.5)	23.5 (11.1) 21 (15.75;31)	0.038*
LEP P2 Amplitude (µV)	29.39 (8.7) 28.5 (22;35.25)	27.55 (14.27) 23 (19.25;40.5)	0.609
CPM (NRS 0–100) (Last minus first)	−0.98 (9.25) 0.00 (−9.25;7.12)	−9.92 (11.32) −10.00 (−17.25; −1.12)	0.006*

Note

Each value is expressed as Mean (*SD*) (first line) and Median (25;75 IRQ) (second line).

CDT, cold detection threshold; WDT, warm detection threshold; CPT, cold pain threshold; HPT, heat pain threshold; NRS, Numerical Rating Scale; LEP, laser‐evoked potentials; CPM, conditioned pain modulation.

* *p* < 0.05.

**FIGURE 1:**
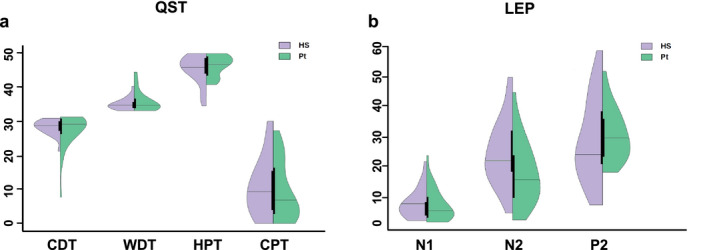
Violin plots of QST (a) and LEP (b) variables by disease status (healthy subjects in purple and NSSI patients in green) with median value and interquartile range. HS, healthy subjects; Pt, patients; CDT, cold detection threshold; WDT, warm detection threshold; CPT, cold pain threshold; HPT, heat pain threshold

The comparison of QST data between patients and control group was not significant for any of the variables explored (Table 1) (Figure 1a). In the individual analysis, QST values, compared with published reference data (Rolke et al. 2006), were within normative ranges.

The N2 amplitude of laser‐evoked potentials and the CPM effect significantly differed between patients and controls (*p* < 0.04; *p* < 0.006).

**FIGURE 2:**
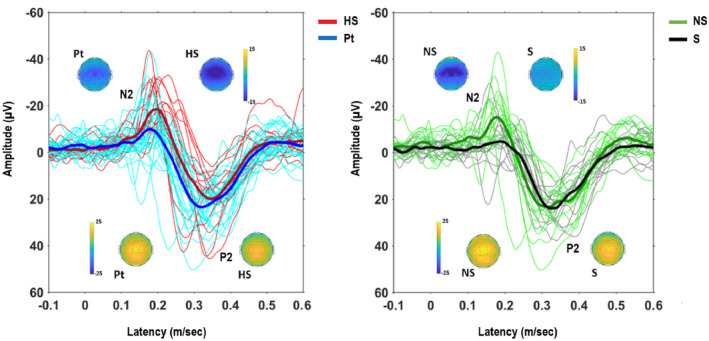
Vertex components and related scalp maps of the evoked potentials (Cz‐Nose recording) after hand stimulation. Superimposed individual traces, grand average in bold. (a) Patients versus healthy subjects; (b) non‐suicidal versus suicidal. HS, healthy subjects; Pt, patients; NS, non‐suicidal; S, suicidal

The N2 amplitude of laser‐evoked potentials was significantly lower in patients compared to healthy subjects. We did not disclose any other significant difference between patients and healthy subjects, in terms of laser pinprick threshold, NRS of pinprick and amplitude of the other components: N1, P2 (Table 1) (Figures 1, 2).

In patients, the pain rating related to the conditioned test stimuli was similar to that related to the unconditioned test stimuli (*p* > 0.8); the CPM protocol therefore yielded a value around 0 (−0.9), thus indicating a deficit in the descending inhibitory pain control (Table 1). In the control group, the CPM protocol variables were compatible with an intact endogenous pain modulation (Table 1).

### 3.3 Patients’ correlation analysis

We found no significant correlation between duration and severity of the disease with thermal and pinprick thresholds, pain ratings and LEPs/CPM variables among the patients’ group.

### 3.4 Seeking a candidate biomarker for suicide: Suicidal versus non‐suicidal group

No significant differences were found regarding psychological variables (Tables 2, 3 and S1), except for a tendency towards significance for the hopelessness scale (BHS) (*p* = 0.082, Table 2) and the personality subfacets anhedonia, impulsivity and risk‐taking of the PID‐5 (*p* = 0.062; *p* = 0.068; *p* = 0.079, Table S1), which resulted increased in the suicidal group. The N2 component of laser‐evoked potentials was significantly lower in suicidal patients than in non‐suicidal (*p* < 0.02 Table 2, Figure 2b).

**TABLE 2:** Univariate analysis for demographical, psychological and neurophysiological data

	Non‐suicidal (14)	Suicidal (16)	*p* value
​Duration (months)	21.43 (22.85) 10.5 (1;44.5)	18.06 (19.97) 12.5 (1.25;29.75)	0.739
R‐NSSI	29.57 (9.62) 32.5 (27.25;35.25)	32 (9.71) 35 (31;37)	0.505
DSHI	4.28 (2.81) 4.5 (1.5;7)	4.4 (2.69) 5 (3;6)	0.912
BHS	11.14 (6.27) 9.5 (5.5;18.25)	15.13 (5.56) 17 (11;19)	0.082
CDI	28.21 (9.49) 29.5 (20.75;38)	31.4 (8.62) 32 (29;37)	0.352
CDT (˚C from baseline)	26.8 (6.2) 28.65 (26.2; 30.3)	28.1 (3.8) 29.4 (25.6; 31.1)	0.506
WDT (˚C from baseline)	36.21 (3.5) 34.85 (33.5; 37.68)	35.48 (2.6) 34.7 (33.8; 36.3)	0.535
CPT (˚C)	8.15 (7.7) 5.75 (1.1; 13.63)	9.9 (9.3) 7.5 (2.8; 20.2)	0.589
HPT (˚C)	46.79 (2.48) 47.2 (45; 48.65)	46.25 (3.5) 46.8 (42; 49.8)	0.639
Laser Pinprick Thr (mj/mm^3^)	79.07 (16.71) 76 (64; 89)	85 (20) 82.5 (76; 89)	0.385
LEP N1 AMP (µV)	4.85 (2.95) 4.25 (2.25; 7.5)	7.4 (6.8) 4.4 (1.6; 14)	0.211
LEP N2 AMP (µV)	21.46 (10.39) 20 (12.75; 27.75)	12.63 (8.9) 11.5 (6; 20.5)	0.020*
LEP P2 AMP (µV)	30.27 (10.45) 28.5 (21.25; 37.5)	28.63 (7.1) 28.5 (22.25; 34.25)	0.282
CPM (NRS 0–100) (Last minus first)	1 (9.4) 0.25 (−6; 8.5)	−2.7 (9) −1.25 (−10; 5)	0.279

Note

Each value is expressed as Mean (*SD*) (first line) and Median (25;75 IRQ) (second line).

R‐NSSI Q, the repetitive non‐suicidal self‐injury questionnaire; DSHI, deliberate self‐harm inventory; BHS, beck hopelessness scale; CDI, child depression inventory. CDT, cold detection threshold; WDT, warm detection threshold; CPT, cold pain threshold; HPT, heat pain threshold LEP, laser‐evoked potentials; CPM, conditioned pain modulation.

* *p* < 0.05.

**TABLE 3:** Association test for binary categorical variables

	Non‐suicidal (14)	Suicidal (16)	*p* value
CRS‐NSSI^†^	7.1%	37.5%	0.086
Cutting	76.9%	100%	0.220
Burning	23.1%	30.8%	1.000
Carving	38.5%	46.2%	1.000
Scratches	61.5%	46.2%	0.694
Bites	46.2%	46.2%	1.000
Sticking pointed objects	61.5%	38.5%	0.433
Head‐banging	69.2%	69.2%	1.000
Hitting	15.4%	38.5%	0.378
Prevent wound healing	38.5%	46.2%	1.000
Acid	0%	0%	–

Note

Each value is expressed as percentage frequencies.

* *p* < 0.05.

^†^ The dichotomization of the severity classes is motivated by the fact that only few cases of class 4 were observed in the sample and no significant difference between class 1 and 2 was detected with a preliminary estimation of the model with a three‐level severity covariate.

### 3.5 Analysis of variance for comparing LEP‐N2 and CPM levels among controls, non‐suicidal and suicidal patients

**FIGURE 3:**
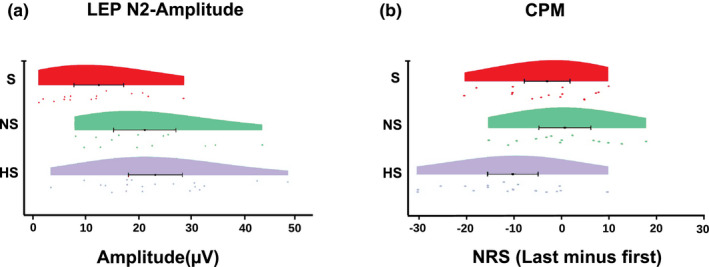
Rainclouds with the 95% CI for the mean of the analysis of variance between subgroups for (a) the LEP N2 amplitude and (b) the CPM paradigm. Each dot represents a single subject. HS, healthy subjects; NS, non‐suicidal; S, suicidal

The analysis of variance (ANOVA) of the N2 amplitude between groups (control, non‐suicidal, suicidal) disclosed a significant difference between control versus suicidal (*p* < 0.003) and non‐suicidal versus suicidal (*p* < 0.02), while no significant difference was found between control versus non‐suicidal (*p* > 0.5) (Figure 3a).

The analysis of variance (ANOVA) of the CPM variable between groups (control, non‐suicidal, suicidal) disclosed a significant difference between control versus suicidal (*p* < 0.04) and control versus non‐suicidal (*p* < 0.004), while no significant difference was found between suicidal versus non‐suicidal (*p* > 0.3) (Figure 3b).

### 3.6 Multivariate analysis

Through the multicollinearity analysis (supplemental material), we specified a logistic regression model with 10 potential predictors of the suicidal risk. The optimal model in terms of Akaike information criterion value was identified with backward selection procedure and the corresponding estimates are shown in Table 4. The LEP N2 amplitude variable was found to be the only statistically significant predictor of suicidal risk (*p* = 0.036). Specifically, the negative estimated coefficient indicated that lower LEP N2 amplitude values in NSSI patients are associated with an increased suicide risk. A borderline result emerged for the effect of the severity class of the NSSI (*p* = 0.062) evaluated through the CRS‐NSSI.

**TABLE 4:** Results of the logistic regression model for the suicide attempt

Predictor	Estimate	95% CI	*p*‐value
LEP N2 Amplitude	−0.117	[−0.250, −0.023]	0.036^a^
CRS‐NSSI: 3–4 verus 1–2	2.339	[0.198, 5.492]	0.062^b^

^a^ Significant result at level <0.05.

^b^ Significant result at level <0.10.

Several goodness‐of‐fit tests were applied to the estimated logistic model (Table S2); they all provided evidence in favour of the model adequacy to the observed data.

**FIGURE 4:**
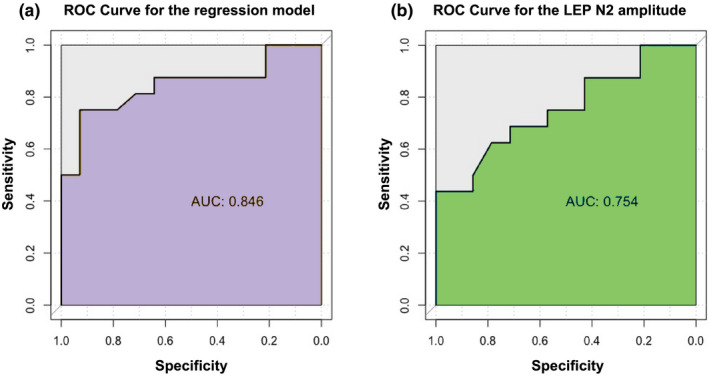
ROC curve of the estimated logistic regression model (a) and the LEP N2 amplitude values (b) for predicting the suicide attempt

The overall performance of the model to predict the outcome was explored with the ROC curve (Figure 4a) and the related accuracy measures. The estimated logistic model exhibited a significant AUC equal to 0.85 (*p* < 0.001) as well as appreciable SE = 75% and SP = 93%. Additionally, the sum of SE and SP is manifestly above 1, namely 1.679, confirming a global good predictive power. PPV and NPV values were, respectively, 92% and 76%.

The accuracy of the sole LEP N2 amplitude value as a biomarker of suicide was also investigated with the construction of the corresponding ROC curve reported in Figure 4b. The overall diagnostic ability of the LEP N2 amplitude as a predictor is quantified by a significant AUC equal to 0.75 (*p* < 0.001). The optimal threshold was 14 (µV), corresponding to SE = 69% and SP = 71%. PPV and NPV were, respectively, 73% and 67%.

## 4 DISCUSSION

Our findings disclosed an abnormal pain modulation in patients with NSSI and a reduced amplitude of the N2 component of laser‐evoked potentials. The latter should be regarded as a potential biomarker of suicidal risk.

### 4.1 PAIN AND NSSI

#### 4.1.1 QST findings and laser‐evoked potentials

The findings of normal thermal pain, laser pinprick thresholds and pain ratings in our patients, compared to healthy subjects, are in contrast with previous studies in patients with self‐injurious behaviours associated with borderline personality disorder (BPD) that had used the cold pressor test (Bohus et al. 2000; Russ et al. 1992), a tonic radiant heat ramp (Kemperman et al. 1997), phasic heat pulses (Schmahl et al. 2004) or mechanical pinprick stimulation (Magerl et al. 2012) to elicit pain. All these studies were conducted in adult patients with BPD and a relatively long history of self‐injurious behaviours. Indeed, an abnormal central pain processing has been observed in several psychiatric conditions (Asmundson & Katz, 2009; Atik et al. 2007; Davis et al. 1979; Singh et al. 2006; Stubbs et al. 2014). Several studies have proved that overlapping neurotransmitter systems (mainly serotonin, noradrenalin and dopamine) (Dayer, 2014; Naliboff and Rudy 2009) as well as overlapping brain networks (Navratilova & Porreca, 2014; Palaniyappan & Liddle, 2012) may underlie both pain and psychiatric disorders. A recent study by Ludäscher and colleagues (2015) specifically explored thermal pain thresholds in adolescents diagnosed with BPD and found that disturbed pain processing is not only a consequence of chronic BPD but is already present in early stages of disease. According to our findings, disclosing normal thermal pain thresholds and pain ratings, together with the lack of correlation with duration and severity of self‐harming, we can conjecture that the increase in pain thresholds observed in BPD patients could be interpreted as a plastic phenomenon due to the underlying personality disorder, rather than the consequence of self‐injurious behaviour and, hence, the result of an adaptive effect.

Laser‐evoked potentials disclosed a significant amplitude reduction of the N2 component in patients group as compared to controls. These findings may seem in contrast with previous results by Schmahl and colleagues using laser‐evoked potentials and showing no general impairment of the spinothalamic pathway in this category of patients (2004). However, the study was conducted in a cohort of adult patients with self‐injury behaviour associated with a defined personality disorder (BPD), therefore not comparable with the adolescent population of our study, diagnosed with NSSI alone. Furthermore, a more in‐depth analysis of our data disclosed that the subgroup of patients with attempted suicide significantly weighted on the difference between patients and controls and that the non‐suicidal population behaved like controls (Figure 3a). Since the study by Schmahl et al. did not provide any information about suicide attempts, we can alternatively hypothesize that their population was mainly composed by non‐suicidal patients.

#### 4.1.2 Conditioned pain modulation

The CPM disclosed a deficit of the endogenous pain modulation in patients with NSSI. The lack of function of the endogenous system seems not to validate the interesting hypothesis proposed by Magerl and colleagues (2012), suggesting that hypoalgesia in BPD self‐harming adults may result from a learning process caused by repeated self‐injury and involving the plasticity of the endogenous pain control. Admittedly, our cohort of adolescent patients, while describing hypoalgesia during self‐harming, did not show the increased pain thresholds observed in BPD adults; therefore, any conclusion may sound speculative. A recent study by Defrin and colleagues (2020) demonstrated an increased function of the endogenous pain modulation system in adult patients with BPD. The lack of differences between patients with BPD and patients with BPD and self‐injury behaviour led the authors to hypothesize that the enhanced pain modulation could be attributed to dissociation, a peculiar feature of BPD. Our findings may rely on a saturation due to the repetition of self‐injurious behaviours. Previous studies on patients with continuous pain stress, such as fibromyalgia (Lautenbacher 1997), irritable bowel syndrome (Wilder‐Smith 2007) and functional abdominal pain (Morris 2016) proved a deficit of the endogenous pain control, possibly explained by a continuous solicitation of the endogenous system, resulting in a lack of function.

Another interesting explanation to our results concerning the CPM deficit should be sought in the theory proposed by Schmahl and Baumgärter (2015) to explain self‐injury behaviour in BPD. They stated that, in healthy subjects, a lesion simultaneously causes stress and pain, where the pain acts as a trigger for further stress. In animals, it is called stress‐induced analgesia, where stress can reduce pain perception. This interaction is altered in many patients with BPD and self‐injury behaviour, where the self‐injurious act is used as a vehicle to reduce inner aversive tension with the side effect of pain, bringing the patient to a lower level of tension (Schmahl and Baumgärter 2015). From this point of view, the effectiveness of the endogenous pain modulation would be counterproductive, reducing the effect of the lesion on the lowering of the tension.

### 4.2 Suicidality and NSSI

The chance to follow our patients for more than 2 years allowed us to register the incidence of attempted suicide and, more importantly, to isolate any biological variables among the ones collected that would allow us to distinguish NSSI from a self‐injury behaviour prodromal to an attempted suicide.

#### 4.2.1 Psycho‐diagnostic evaluation

The psychopathological instruments used in the study did not clearly distinguish between suicidal and non‐suicidal patients. A recent meta‐analysis on the correlates of suicide attempts (SA) among self‐injurers (Victor & Klonsky, 2014) found that the strongest correlate of SA history was suicidal ideation, while other NSSI specifiers, including NSSI frequency, number of NSSI methods and hopelessness, were weaker predictors. Indeed, although our findings only approached the statistical significance, we found overlapping results in our cohort of patients.

NSSI severity evaluated through the clinician‐rated severity of non‐suicidal self‐injury (CRS‐NSSI), proved to be a weak predictor of suicide: patients with grade 3 and 4, namely those with moderate to severe grade of NSSI, had a higher risk of suicide attempt during the study period.

The Bech Hopelessness Scale, evaluating the presence and degree of Hopelessness resulted increased in suicidal patients as compared with non‐suicidal (*p* < 0.1). Although elevated hopelessness can be considered a common trait to all self‐harming, many authors pointed out its predictive role for suicide (Klonsky et al. 2016).

Regarding Personality inventory data, we found that three subfacets approached significance: anhedonia, impulsivity, risk‐taking. The anhedonic trait has been observed in subjects with suicidal behaviour including adolescents (Brausch & Gutierrez, 2010; Muehlenkamp & Gutierrez, 2007) and is considered an important risk factor for suicidality in subjects with different psychiatric conditions (Bradley et al. 2015; Fawcett et al. 1990; Kollias et al. 2008; Nock & Kazdin, 2002; Spijker et al. 2010; Winer et al., 2014).

Several studies have also reported impulsivity and risk‐taking traits as predictors of suicidal attempt especially among adolescence, where completed suicide often occurs suddenly, unexpectedly and impulsively (Brodsky et al. 1997; Hawton et al. 1999; Mann et al., 1999; Nock and Kes 2006; Klonsky & May, 2010).

In light of the results of PID‐5 and clinical assessment, our sample does not seem to show a clear trajectory oriented towards BPD, thus suggesting that NSSI may have a significant clinical and diagnostic heterogeneity and is not just an expression of underlying or prodromal BPD. It is, therefore, important to recognize NSSI as an independent diagnosis with peculiar features.

#### 4.2.2 Neurophysiological evaluation

The most interesting data we obtained are the presence of a significant reduction of the N2 component of laser‐evoked potentials’ amplitude in subjects who attempted suicide compared to subjects who maintained a non‐suicidal self‐injury behaviour. This finding was further supported by an accurate multivariate analysis which found the LEP N2 amplitude variable to be the only statistically significant predictor of suicidal risk with lower N2 amplitude values in NSSI patients associated with an increased probability to attempt the suicide.

The N2 component of laser‐evoked potentials generated at the level of the anterior cingulate and of the anterior mid‐insula (Garcia‐Larrea, 2003) is the only component among the three explored (N1, N2, P2) that resulted predictive for suicide. The integrity of the other two components points to a predominant impairment of the anterior insula/anterior cingulate or to a reduced connectivity between these regions and the posterior insula in NSSI patients attempting suicide and, therefore, to a predominant impairment of the affective‐motivational aspect of pain (Brooks and Tracy 2005; Garland, 2012).

Indeed, previous functional neuroimaging and post‐mortem studies in patients with suicidal behaviour (see van Heeringen & Mann, 2014 for a comprehensive review) disclosed structural and functional changes of the anterior cingulate and the insula associated with suicide (van Heeringen & Mann, 2014; Tanaka et al. 2004; Giakoumatis et al. 2013; Pan et al. 2011 and 2013; Dombrovski et al. 2013; Benedetti et al. 2011).

On the other hand, both the lateral and medial pain systems have been shown to be dysfunctional in patients with major psychiatric disorders associated with psychosis (Minichino et al. 2016). Indeed, the medial, affective‐motivational ‘matrix’ of pain perception is also part of the so‐called ‘salience network’, a neural network that functions to segregate the most relevant among internal and extra personal stimuli to guide behaviour and a dysfunction of this network has been related to the expression of psychotic symptoms (Palaniyappan & Liddle, 2012).

Given these considerations, it is possible that the N2 component of laser‐evoked potentials could represent an unspecific measure of salience circuit dysfunction and, therefore, not a specific biomarker for suicide, but rather an index of impairment of subjective evaluation of one's affective state and of internalization of perceptions of oneself by others, a common finding in several psychiatric disorders (New et al., 2008). Finally, LEP N2 component is also sensitive to changes in attention, both general and selective (Franz et al. 2015; Legrain et al. 2002), and its reduction could reflect a lack of attentional drive, which is itself linked to suicidality (Fernández‐Sevillano et al. 2021). In this case, the N2 component would be just an intermediate marker. Admittedly, we did not perform a specific task to monitor attention, besides asking each subject to mentally count the stimulations perceived to maintain a steady vigilance/attention (Cruccu 2008) nor we applied specific psychological tests to evaluate attention deficits. Future studies are needed, specifically testing the influence of attentional factors, to clarify to what extent the N2 reduction could be explained by attention.

However, a biomarker can demonstrate good clinical utility because it is able to stratify patients into clinically meaningful categories, with a high sensitivity and specificity, without directly reflecting the mechanisms that give rise to a given clinical condition, as the patterns of neural activation that allow discrimination between conditions might be entirely epiphenomenal (Mouraux and Iannetti 2018).

Although not specific, the amplitude of the N2 may be considered a candidate biomarker due to the rapidity and the non‐invasiveness of the examination and the good sensitivity in identifying a population at risk among self‐harming adolescents. The area under the curve of ROC disclosed a sensitivity around 70% below a cut‐off of 14 μV.

### 4.3 LIMITATIONS

The results of this study should be considered in the light of several limitations. First, the lack of information about the reproducibility of our findings: Further studies are needed to confirm the N2 reduction in suicidal patients among adolescent self‐harmers. Without replications, the potential role of the N2 component as a biomarker of suicide cannot be determined. Second, we cannot rule out the possibility that the medications used by NSSI patients affected the results obtained (Mucci et al. 2006). However, recent observations demonstrated the absence of analgesic properties of psychiatric drugs (Jochum et al. 2006; De Tommaso et al. 2011; Potvin et al. 2008) and a previous study by our group on LEPs in psychiatric patients found no effect of medication (Minichino et al. 2016). Regarding the comparison between suicidal and non‐suicidal patients, the drug intake was balanced between the two populations.

## 5 CONCLUSIONS

Our findings indicate that NSSI is associated with an abnormal pain processing not related to an over function of the endogenous pain control, which, on the contrary, shows a dysfunction possibly resulting from a saturation due to the repetition of self‐injurious behaviours. Among NSSI patients, the amplitude of the LEP N2 component could be a candidate biomarker for suicide, even stronger when associated with a high severity of disease assessed by the CRS‐NSSI.

Further studies are needed to replicate our data and test for specificity. Since the presence of this neurophysiological alteration in adolescents with onset of NSSI behaviours offers the chance to use a non‐invasive test to identify a subgroup of patients with a higher risk of suicide among self‐harming adolescents, we encourage further research to validate our data and possibly introduce laser‐evoked potentials in the diagnostic assessment of these patients.

## CONFLICT OF INTEREST

Prof. Giorgio Cruccu declares to have a consulting contract with Angelini and Byogen and a research grant and personal fees with Alfasigma. Prof. Andrea Truini declares to have a relationship with Alfasigma, Angelini, Pfizer and Grunenthal as speaking fees. The other authors have nothing to declare.

## AUTHORS CONTRIBUTION

CL, SG and AT designed the experiment; CL, SG, AT, SM, VM, LB, CP and MF collected the data; CL and CM analysed the data; CL, SG, AT, GC, MF drafted the manuscript and gave final approval.

## Supporting information

Table S1

Table S2

Supplementary Material
