# Supplementary material for: Dissecting pain processing in adolescents with Non‐Suicidal Self Injury: Could suicide risk lurk among the electrodes?
Source: Eur J Pain. 2021 May 31;25(8):1815–28. doi: 10.1002/ejp.1793 (PMC8453562; doi:10.1002/ejp.1793)
Supplement: Supplementary file 1 — Table S1 [file EJP-25-1815-s002.docx]

| **PID-5 trait scale** | **Non suicidal (14)** | **Suicidal (16)** | **P value** |
| --- | --- | --- | --- |
| **Anxiousness** | 2.03 (0.52)  2.2 (1.58;2.2) | 2.16 (0.57)  2. (1.75;2.72) | 0.554 |
| **Emotional Lability** | 2.21 (0.73)  2.36 (1.7;2.78) | 2.36 (0.53)  2.50 (1.4;3) | 0.560 |
| **Hostility** | 1.61 (0.63)  1.7 (1;2.2) | 1.56 (0.79)  1.5 (0.77;2.3) | 0.853 |
| **Perseveration** | 1.61 (0.37)  1.67 (1.33;1.89) | 1.34 (0.55)  1.44 (0.86;1.78) | 0.161 |
| **Restricted Affectivity** | 1.32 (0.68)  1.50 (0.64;1.82) | 1.06 (0.36)  1 (0.6;1.7) | 0.258 |
| **Depressivity** | 1.88 (0.69)  1.82 (1.6;2.4) | 2.25 (0.80)  2.46 (0.3;2.9) | 0.220 |
| **Separation Insicurity** | 1.70 (0.85)  1.85 (0.9;2.53) | 1.57 (0.61)  1.43 (0.6;2.6) | 0.741 |
| **Suspiciousness** | 1.6 (0.41)  1.56 (1.2;1.96) | 1.80 (0.57)  1.78 (1.57) | 0.363 |
| **Submissiveness** | 1.30 (0.76)  1.25 (0.8;1.96) | 1.66 (0.78)  2 (0;2.7) | 0.258 |
| **Anhedonia** | 1.73 (0.54)  1.75 (1.38;1.88) | 2.19 (0.59)  2.37 (0.7;3) | 0.062* |
| **Intimacy Avoidance** | 1.46 (0.91)  1.81 (0.42;1.89) | 1.06 (0.36)  1 (0.6;1.7) | 0.256 |
| **Withdrawal** | 1.31 (0.9)  1.15 (0.5;2.32) | 1.15 (0.54)  1.25 (0.2;2) | 0.547 |
| **Attention Seeking** | 1.14 (0.86)  0.87 (0.56;1.6) | 1.51 (0.58)  1.70 (0.2;2.3) | 0.453 |
| **Callousness** | 0.66 (0.51)  0.57 (0.2;0.9) | 0.88 (0.82)  0.56 (0;2.5) | 0.837 |
| **Deceitfulness** | 0.88 (0.67)  0.80 (0.32;1.19) | 0.71 (0.41)  0.85 (0;1.4) | 0.807 |
| **Grandiosity** | 0.37 (0.38)  0.18 (0.04;0.62) | 0.97 (0.66)  1 (0;2) | 0.555 |
| **Manipulativeness** | 0.74 (0.67)  0.70 (0.2;1.12) | 0.26 (0.47)  0 (0;1.5) | 0.960 |
| **Distractibility** | 2.1 (0.71)  2.05 (1.6;2.75) | 0.79 (0.79)  0.70 (0;2.2) | 0.491 |
| **Impulsivity** | 1.89 (0.76)  1.83 (1.12;2.6) | 1.90 (0.76)  2 (0.4;2.9) | 0.068* |
| **Irresponsibility** | 1.52 (0.63)  1.71 (1;1.94) | 1.32 (0.76)  1.33 (0;2.5) | 0.164 |
| **Perfectionism** | 1.12 (0.43)  1.05 (0.8;1.37) | 1.18 (0.51)  1.14 (0.1;2) | 0.648 |
| **Rigid Perfectionism** | 1.84 (0.46)  1.95 (1.62;2.17) | 1.23 (0.85)  1.35 (0;3) | 0.519 |
| **Risk Taking** | 1.68 (0.52)  1.60 (1.2;2.2) | 1.68 (0.81)  1.60 (0;2.8) | 0.079* |
| **Eccentricity** | 1.70 (0.6)  1.66 (1.38;1.9) | 1.32 (0.55)  1.30 (0.2;2.1) | 0.964 |
| **Perceptual Dysregulation** | 1.28 (0.50)  1.41 (0.94;1.62) | 1.73 (0.56)  1.66 (1;3) | 0.223 |
| **Unusual beliefs** | 1.08 (0.7)  1.09 (0.5;1.62) | 1.62 (0.71)  1.71 (0.6;2.7) | 0.750 |

**Tab.S1 Univariate analysis of the personality inventory data (PID)**

Each value is expressed as Mean (SD) (1st line) and Median (25;75 IRQ) (2nd line)

**P<0.05; *P<0.1
