# Supplementary material for: Dissecting pain processing in adolescents with Non‐Suicidal Self Injury: Could suicide risk lurk among the electrodes?
Source: Eur J Pain. 2021 May 31;25(8):1815–28. doi: 10.1002/ejp.1793 (PMC8453562; doi:10.1002/ejp.1793)
Supplement: Supplementary file 2 — Table S2 [file EJP-25-1815-s001.docx]

| **Goodness-of-fit test** | ***p*-value** |
| --- | --- |
| **Hosmer-Lemeshow test** | 0.240 |
| **Osius-Rojek test** | 0.478 |
| **le Cessie-van Houwelingen-Copas-Hosmer test** | 0.816 |

**Tab.S2 Goodness-of-fit tests for the logistic regression model for the suicide attempt**

p-value < 0.05 is evidence of a poor fit
