# Supplementary material for: Dissecting pain processing in adolescents with Non‐Suicidal Self Injury: Could suicide risk lurk among the electrodes?
Source: Eur J Pain. 2021 May 31;25(8):1815–28. doi: 10.1002/ejp.1793 (PMC8453562; doi:10.1002/ejp.1793)
Supplement: Supplementary file 3 — Supplementary Material [file EJP-25-1815-s003.docx]

**Supplemental Material for statistical methods**

The extent and issues of multicollinearity were addressed with the evaluation of the Pearson correlation matrix, the related tests of significance, the variance inflation factor (VIF), the Farrar-Glauber test, and the condition index (Belsley, Kuh and Roy 1980, Midi et al. 2010). Then, the principal component analysis (PCA) was implemented to detect the most informative covariates in such a way to preserve the most part of the variability of the data (Hotelling 1933, Abdi and Williams 2010).

To test the relationships among explanatory variables, we worked separately on the neurophysiological and psychological measures since, for both groups, the multicollinearity problem was strongly suspected. To facilitate the interpretation of the Pearson correlation matrices, we adopted the correlograms reported in **Figure S1** to display the significant coefficients.


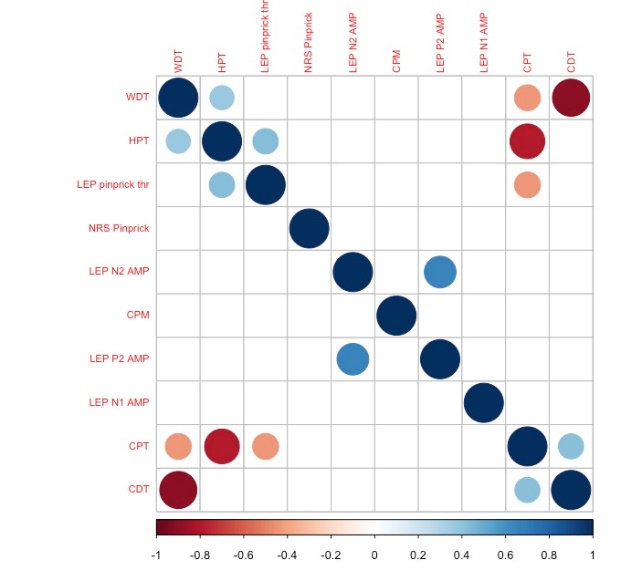

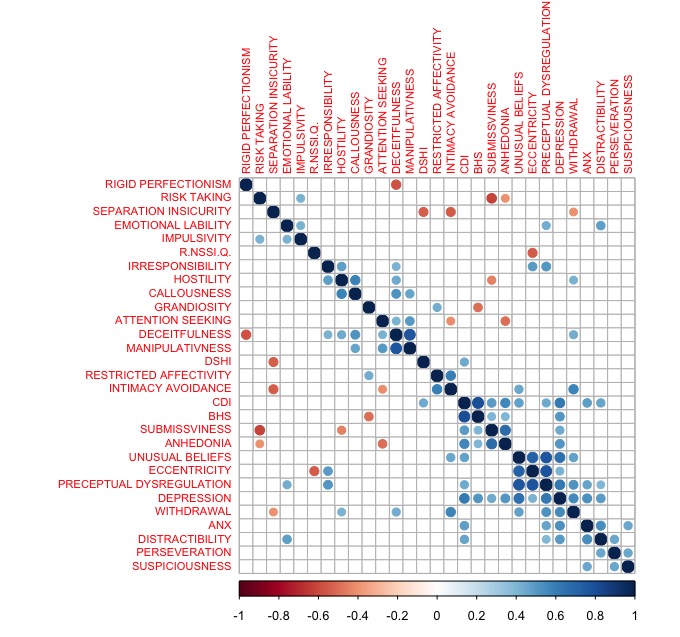


Figure S1. Correlograms of Pearson’s correlation coefficients for neurophysiological (left) and psychological (right) variables. The area and color of the circles is proportional to the magnitude of the correlation coefficient between each pair of numeric variables. Non-statistically significant coefficients are indicated with blank cells.

For the neurophysiological measures, the examination of the correlation matrix revealed the presence of highly and significantly correlated predictors, in particular for the pairs (WDT, CDT) with Pearson’s coefficient r = -0.90, (HPT, CPT) with r = -0.79 and (Lep N2 Amp, Lep P2 Amp) with r = 0.65 (**Figure S1,left**). This contributed to the multicollinearity phenomenon, which is detected by the specific diagnostics illustrated in **Table 1**. By considering a correlation coefficient above 0.65 as a signal of a sizeable information overlapping, a recommended remedy to address the redundancy issue is keeping in the study only one variable per each pair of correlated predictors. Specifically, among the highly correlated variables, we kept WDT, HPT and Lep N2 Amp. With this selection, no further warning was obtained from the multicollinearity check.

The multicollinearity analysis was more challenging for psychological variables, due to the large number of collected measures expressing the multiple facets of complex and interrelated individual conditions. We decided to rule out composite indices (PID-domains) *a priori* because they would certainly have been closely related to the simple indices they are made up of and they would not have added new information. The correlation matrix highlighted the presence of groups of significantly correlated variables (r>0.65) (**Figure S1, right**). Unlike what has been done for the neurophysiological pair of variables, dealing with clusters, we considered too arbitrary to rule out one single variable per each cluster of correlated predictors. Notwithstanding the attempt to drop some variable (based on the univariate analysis results), the multicollinearity indices values remarkably exceeded the reference threshold.

**Tab.1 Multicollinearity diagnostics for the neurophysiological variables**

*VIF>5 and a p-value<0.05 for the Farran-Glauber test are indicators of multicollinearity*

Therefore, to adress the strong redundancy of the psychological factors we decided to use the Principal Component analysis (PCA), a multivariate strategy for dimensional reduction used to solve the problem of multicollinearity.

By inspecting the correlations between the covariates and each principal component (**Figure S2**), this approach allowed us to isolate two main independent factors: PC1 including the strongly correlated predictor CDI and BHS (r= 0.8) and PC2 including R.NSSI.Q, that together explained almost all the variability (91%: PC1 50% and PC2 41%) of the entire set of psychological measures. Given the strong correlation between CDI and BHS, we decided to keep BHS, based on the results of the univariate analysis.


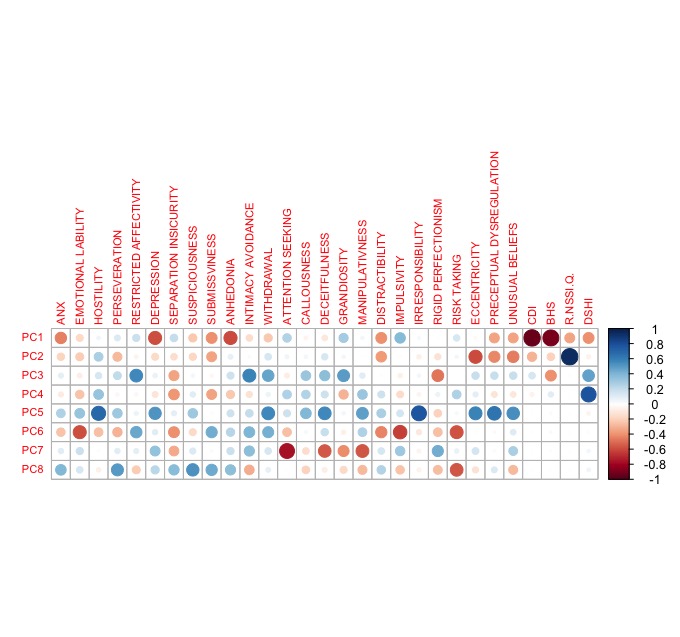


*Figure S2. Correlograms of the psychological variables with the first 5 principal components. The area and color of the circles is proportional to the magnitude of the correlation coefficient between each pair of numeric variables.*

Finally, we included in the logistic regression model the neurophysiological (WT, HPT, Lep pinprick threshold, NRS pinprick, Lep N1 amp, Lep N2 amp and CPM) and psychological (R.NSSI.Q and BHS) variables, plus duration and severity of disease.
